# An Improved Method of Plantar Neurotomy1Read before the Thirty-second Annual Meeting of the U. S. Veterinary Medical Association, Des Moines, Iowa, September, 1895.

**Published:** 1895-10

**Authors:** M. H. McKillip

**Affiliations:** Chicago, Ill.


### AN IMPROVED METHOD OF PLANTAR NEUROTOMY.[1]

BY M. H. McKILLIP, M.D., V.S.,
CHICAGO, ILL.

It is well known to all practitioners who perform a large number of the operation of plantar neurotomy that its practical result is sometimes unsatisfactory, on account of an early uniting of the nerve-ends, with subsequent recurrence of feeling and lameness, no matter how long a section of nerve may have been removed. To overcome this annoying result I have experimentally tried to perfect the modus operandi in different ways, and the result has been so satisfactory that I think it expedient to report it for the benefit of those who are called upon to perform this operation.

As the technique of the operation is familiar to you I shall only refer to the difference of my methods. First of all, I have devised a certain preparation of the limb for the operation, which is quite essential. Three days before operating a cantharides blister is applied over the seat of operation. This is washed off after twenty-four hours, the cuticle removed, and vaseline applied to the part. The object of this procedure is to secure a clean field for operation and rapid union. The blister removes the hair and cuticle, doing away with the hair-stubbles always left by the clipper, which are an irritating agent. It leaves a clean, smooth surface, and prepares, as nearly as possible, for an aseptic operation and dressing. It excites inflammation of the skin, and the slight swelling and increased circulation promote a union by first intention, a result seldom obtained in the old methods, especially in animals with a thin skin. There

[1] Read before the Thirty-second Annual Meeting of the U. S. Veterinary Medical Association, Des Moines, Iowa, September, 1895.

is a tendency to produce a little more hemorrhage during incision, but if any should object to this it is easily prevented by the application of a simple tourniquet.

The operation itself is performed as usual until the nerve has been dissected and cut on its upper end. Then it is dissected downward to a length of about one and a half inches and firmly drawn up with forceps by an assistant, while the operator applies a silk ligature to the lowest part of the distal end, tying it as securely as possible with the surgeon's knot. The dissected nerve-trunk is cut above the ligature, and the silk is allowed to hang out of the lower part of the incision to the length of about one inch. No stitches being used, a cotton bandage saturated with a solution of bichloride of mercury is applied to the fetlock, which draws firmly together the edges of the incision. Over this is again laid an antiseptic flannel bandage. Within three days after the operation the bandages are removed, when the wound will be found to have united by first intention, with the cxception of the lower end, out of which the ligature is now carefully and easily removed. When the ligature comes off the nerve-end is cicatrized, a granulating process is obviated, and the union of the nerve-ends made thus impossible. The parts are dressed and the bandages replaced in order to support the newly-united tissues. Shower-baths for a few days have been found of good service, and in from five to ten days the horse is again ready for work, a result which can hardly be obtained by the old methods.

Of about one hundred horses so operated on by me since February last, no report has been made so far of an unsatisfactory result, and all those cases which could be kept under observation have proved a decided success, not only from a surgical view but also from the practical results obtained.

---

THE election of three new vice-presidents, namely, Drs. Osgood, Lyford, and R. H. Harrison, brings with the newly elected Secretary, Sesco Stewart, much new blood into the official family of the U.S.V.M.A. With the appointment of ex-Secretary Pearson, Dr. D. E. Salmon, and the reappointment of Dr. Thomas B. Raynor to the Executive Board the work of the Association should progress professionally, geographically, and officially.

